# Impact of light therapy on rotating night shift workers: the EuRhythDia study

**DOI:** 10.1007/s00592-022-01956-2

**Published:** 2022-08-31

**Authors:** Stefano Rizza, Alessio Luzi, Maria Mavilio, Marta Ballanti, Arianna Massimi, Ottavia Porzio, Andrea Magrini, Juliane Hannemann, Rossella Menghini, Jonathan Cridland, Bart Staels, Peter J. Grant, Rainer H. Boger, Nikolaus Marx, Massimo Federici

**Affiliations:** 1 Department of Systems Medicine, University of Rome Tor Vergata, Via Montpellier 1, 00133 Rome, Italy; 2 Department of Experimental Medicine, University of Rome Tor Vergata, Via Montpellier 1, 00133 Rome, Italy; 3 Department of Biomedicine and Prevention, University of Rome Tor Vergata, Via Montpellier 1, 00133 Rome, Italy; 4 Institute of Clinical Pharmacology and Toxicology, University Medical Center Hamburg-Eppendorf, Hamburg, Germany; 5 LUMIE, Cambridge, UK; 6 INSERM, CHU Lille, Institut Pasteur de Lille, University of Lille, U1011, EGID, 59000 Lille, France; 7 Department of Cardiology, University Medical Center Aachen, Aachen, Germany; 8 Leeds Institute of Cardiovascular and Metabolic Medicine, University of Leeds, Leeds, UK

**Keywords:** BMAL1, Clock genes, Diabetes, Light therapy, Night shift work, REV-ERBs

## Abstract

**Aims:**

Disturbances in circadian rhythms may promote cardiometabolic disorders in rotating night shift workers (r-NSWs). We hypothesized that timed light therapy might reverse disrupted circadian rhythms and glucose intolerance observed among r-NSWs).

**Methods:**

R-NSWs were randomly assigned to a protocol that included 12 weeks on followed by 12 weeks off light therapy (*n* = 13; 6 men; mean age, 39.5 ± 7.3 years) or a no-treatment control group (*n* = 9; 3 men; mean age 41.7 ± 6.3 years). Experimental and control participants underwent identical metabolic evaluations that included anthropometric, metabolic (including oral glucose tolerance tests), lipid, and inflammation-associated parameters together with an assessment of sleep quality and expression of circadian transcription factors REV-ERBα and BMAL1 in peripheral blood mononuclear cells (PBMCs) at baseline, 12 weeks, and 24 weeks of the protocol.

**Results:**

Twelve weeks of warm white-light exposure (10,000 lx at 35 cm for 30 min per day) had no impact on sleep, metabolic, or inflammation-associated parameters among r-NSWs in the experimental group. However, our findings revealed significant decreases in REV-ERBα gene expression (*p* = 0.048) and increases in the REV-ERBα/BMAL1 ratio (*p* = 0.040) compared to baseline in PBMCs isolated from this cohort. Diminished expression of REV-ERBα persisted, although the REV-ERBα/BMAL1 ratio returned to baseline levels after the subsequent 12-day wash-out period.

**Conclusions:**

Our results revealed that intermittent light therapy had no impact on inflammatory parameters or glucose tolerance in a defined cohort of r-NSWs. However, significant changes in the expression of circadian clock genes were detected in PBMCs of these subjects undergoing light therapy.

## Introduction

Many workers in Western societies perform night shift work. One recent review reported that as many as 20% of the working population in Europe is engaged in some type of shift work schedule [1]. Among those employed in the health care sector, this percentage increases to ~ 45% [1, 2]. Biological rhythms may not be well-tuned to or capable of rapid adaptation to changes in these environmental cycles and reduced sleep; frequent time shifts may predispose workers to poor metabolic health [3]. Results from recent epidemiological studies have suggested that rotating night shift work is associated with a higher risk of developing type 2 diabetes (T2D), increased inflammation, and cardiovascular disease [4–7]. Even when the diet is controlled, night-shift workers (NSWs) exhibit poorer metabolic health than those whose work takes place during daytime hours [8]. As but one example, results from the Nurse Health Study II revealed that participants who reported sleep difficulties when working on rotating night shifts had an increased risk of developing T2D [9]. Our group recently reported subclinical abnormalities in HbA1c and changes in expression of circadian clock genes among current NSWs compared to former NSWs and individuals who work during daylight hours only [10]. Prolonged exposure to artificial light also contributes to the disruption of the internal clock. Several studies have revealed that exposure to artificial light at night promoted significant metabolic disturbances in experimental animal models [11, 12]. Furthermore, the results of one recent study revealed that daylight was more effective than exposure to artificial light at night (LAN) for suppression of melatonin excretion in urine. These results suggest that there may be other, as yet unidentified biomarkers that might be used to identify subjects who are sensitive to the negative effects of rotating shift work and LAN [13]. Thus, we performed a small hypothesis-generating study aimed at analyzing the impact of warm white light exposure on the expression of circadian clock genes and several characterized inflammatory and metabolic biomarkers in a cohort of rotating NSWs.

## Methods

### Light therapy protocol

A randomized open-label clinical trial that included a 12-week session of light intervention followed by a 12-week off-protocol period was performed at Policlinico Tor Vergata between 2013 and 2015 as part of the EuRhythDia project. The EuRhythDia consortium investigated potential disruptions of the circadian clock in rotating NSWs and characterized the impact of light therapy. The overall goal was to determine whether this intervention might improve insulin sensitivity and limit inflammation in individuals belonging to high-risk groups. Participants were active rotating NSWs working a shift schedule that included four to as many as seven 12 h overnight shifts nights per month followed by two days off for a minimum of two years. Exclusion criteria included a diagnosis of diabetes, liver disease, renal insufficiency, heart failure, coagulopathy, or any other severe systemic disease. Subjects were also excluded if they had a history of cancer in any form, positive blood tests for human immunodeficiency virus, hepatitis B, and/or hepatitis C, and/or if they had taken melatonin supplements within four weeks before beginning the study. In women, the study was initiated during the early follicular phase of the menstrual cycle. Participants provided written consent after receiving detailed information about the study protocol. The Tor Vergata University Ethics Board approved this study (decision #EU-FP3:125/13). The investigations reported in this study were carried out following the principles of the Declaration of Helsinki (revised in 2000).

Twenty-eight (*n* = 28) subjects were recruited and randomized on a one-to-one basis to the light intervention therapy (*n* = 14) or control groups (*n* = 14). Upon commencing the study and after the first and second 12-week periods as described, anthropometric data and 12-h fasting blood samples were collected from all study participants.

Plasma glucose levels were measured in vitro by an enzymatic test (automated chemistry clinical analyzer, Roche). Serum insulin levels were determined using an electrochemiluminescence assay (Eclia, Roche). A 75 g oral glucose tolerance test (OGTT) with sampling at 0, 30, 60, 90, and 120 min for plasma glucose and serum insulin levels was performed for each subject.

### Real-time (RT)-polymerase chain reaction (PCR)

Approximately 20 ml samples of whole blood were drawn from each subject at each timepoint. Approximately eight ml of blood drawn into a Vacutainer with sodium citrate anticoagulant (Becton–Dickinson) was used to isolate peripheral blood mononuclear cells (PBMCs) as previously described [10, 14]. Single-stranded cDNA was synthesized from 2 μg of total RNA using a High-Capacity cDNA Archive Kit as per the standard protocol (Applied Biosystems, Foster City, CA, USA). Fifty nanogram samples of cDNA were amplified by RT-PCR to assess the expression of circadian clock (REV-ERBα and BMAL1) and inflammatory (IL-1β and IFN-γ) genes using an ABI PRISM 7500 System. Results were normalized to 18S rRNA as the endogenous control.

### Assessment of sleep quality

Perception of sleep during study protocol was determined using the Pittsburgh Sleep Quality Index (PSQI), which is a validated scale that can be used to identify elements of sleep over a previous 30-day period [10]. Briefly, the questionnaire explores seven different components of sleep, including sleep latency, sleep duration, habitual sleep efficiency, sleep disturbance, use of sleep medication, daytime dysfunction, and subjective sleep quality. The sum of scores of these seven components provides a global PSQI score that ranges from 0 to 21 points, with a score of 5 or greater indicative of poor sleep quality. We used this information to dichotomize the scores [good sleepers (score < 5) vs poor sleepers (score ≥ 5)] in our analysis.

### Light therapy

**Fig. 1:**
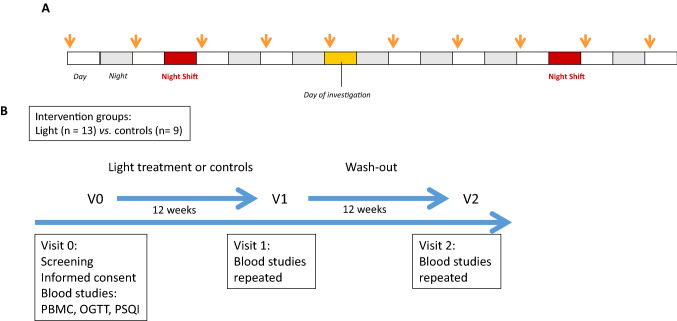
**A** Schedule used for light therapy. Each participant was provided with 1 h of 10,000 lx (see arrows) within the first three hours after awakening. **B** Study protocol used for light therapy and controls.

Subjects randomized to the light therapy group maintained their standard habits (i.e., diet, exercise activity) and sleep–wake patterns on non-night shift days as they had before the study period. Participants were exposed to bright light (10,000 lx at a distance of 35 cm for 30 min) every morning at home between 06:00 and 09:00 h except on those days that immediately followed a night shift (Fig. 1). The subject was allowed to move away from the device briefly, but the total duration of the exposure was a full 30 min. A portable panel Lumie Brazil Seasonal Affective Disorder (SAD) Light (dimensions 50 × 31 × 15 cm, weight 2.85 kg; Lumie, Cambridge, UK) was used as a light source. This SAD Light produced white light with a warm tone at the aforementioned intensity using three 36 W broad spectrum light bulbs. It was not necessary to look at the screen for the entire 30 min, although the participants were instructed to allow light to enter freely into both eyes. At the end of the trial, a questionnaire was provided to each participant in the light therapy group to calculate the extent of overall compliance.

### Statistical analysis

Clinical characteristics were reported as means and standard deviations (SDs) or frequencies and percentages for continuous and categorical variables, respectively. Normal distributions of all continuous variables were confirmed by the Kolmogorov–Smirnov test. Baseline characteristics were compared between groups using the Mann–Whitney U test or Fisher’s exact test. Intergroup comparisons of continuous variables measured at different visits were compared by an analysis of covariance (ANCOVA) with baseline values (at V0) used as a covariate. Within-group comparisons of values identified at V1 *versus* V0 and V2 *versus* V1 were performed using general linear models for repeated measures. *p* values < 0.05 were considered statistically significant. All analyses were performed using SPSS software program version 19.0 for Windows.

## Results

The flowchart for the clinical protocol is shown in Fig. 1. As shown, we used a light treatment protocol used to treat seasonal affective disorders to determine its impact on glucose metabolism and circadian markers in a cohort of rotating NSWs.

During study protocol, no participants changed ordinary diet and physical activity. This point is crucial since intensive workout or diet changing may lead to an improving of reported quality of sleep [15]. Participants who reported only limited compliance (i.e., < 50%; *n* = 1) with the light therapy protocol were excluded from the data analysis. We also excluded five subjects in the control group who did not begin the study protocol after providing informed consent. Thus, 13 participants assigned to the light therapy group and nine control participants completed the study protocol and were included in the final data analysis. Three physicians (A.L., S.R., and M.B.) independently recruited and supervised the test subjects.

**Fig. 2:**
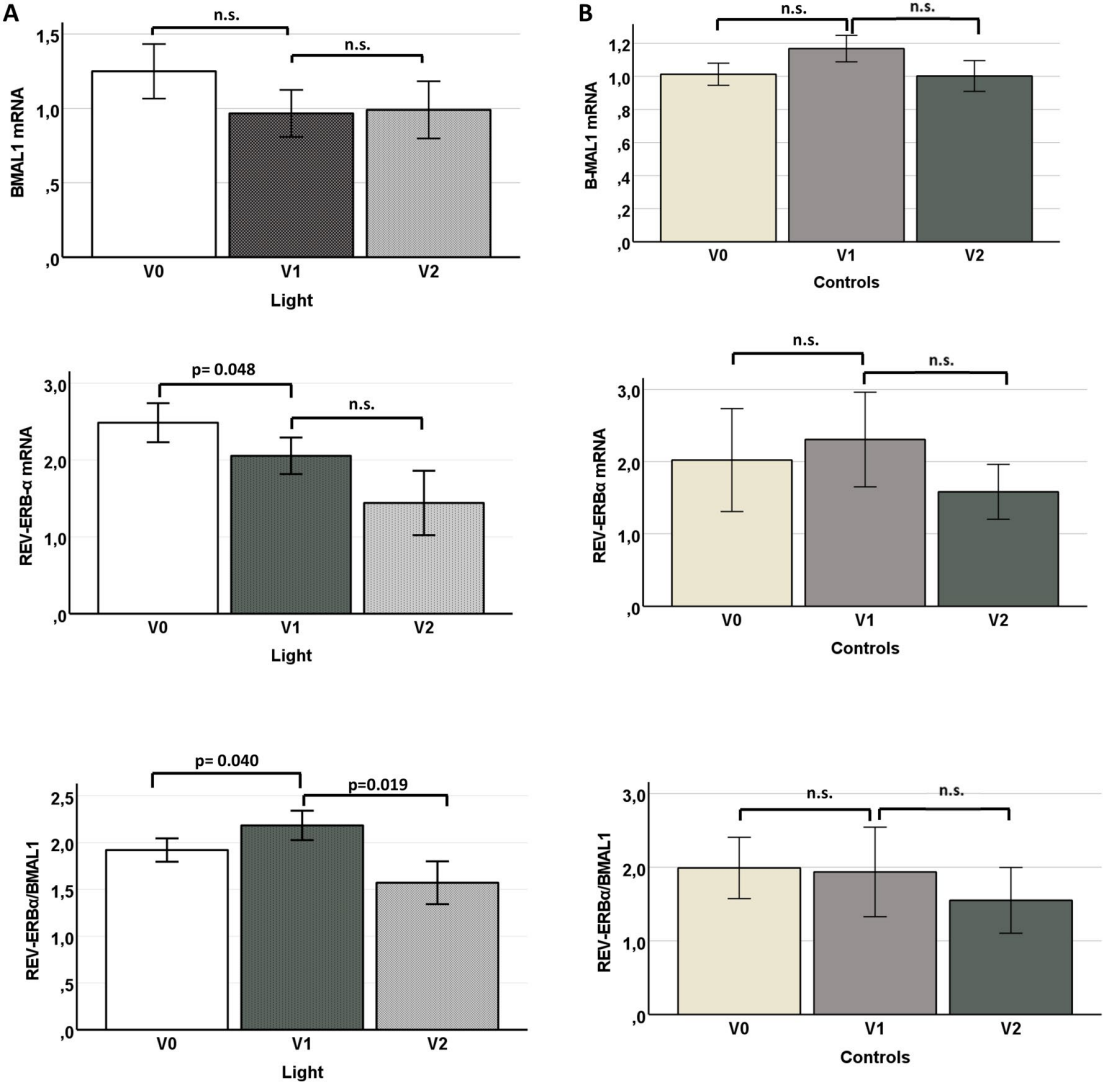
Differential expression of clock genes BMAL1 and REV-ERBαin PBMCs from r-NSWs **A** treated with light therapy or **B** controls; V0, visit 0; V1, visit 1; V2, visit 2

Notably, no participants reported any of the side effects of light therapy previously reported in the literature [16], including headache, eyestrain, nausea, insomnia, and hyperactivity. None of the outcome measures changed in the control group (Table 1). Similarly, none of the metabolic parameters, including lipids, inflammation, glucose tolerance, static (HOMA IR), and dynamic (AUC) glucose and insulin levels or blood pressure underwent significant change in those undergoing light therapy at any of the time points during the study period. Of note, we observed no significant modification of the ratio of bad/good sleepers at any time point during the protocol. Moreover, all of the rotating NSWs had fasting glucose and Hb1Ac levels within normal limits at all times during this study. However, the quantitative evaluation of gene expression in PBMCs from participants treated with light therapy revealed significantly diminished levels of REV-ERBα mRNA compared to baseline (V0 at 2.48 ± 1.54 *versus* V1 at 2.07 ± 0.70, *p* = 0.048). An additional decrease in REV-ERBα mRNA was detected after the 12-week off period (V1, 2.07 ± 0.70 versus V2, 1.47 ± 0.96, *p* = 0.074; Table 1 and Fig. 2). By contrast, expression levels of BMAL1 mRNA were not affected by light treatment. The REV-ERBα/BMAL1 ratio increased significantly at the first time point immediately after completion of the light therapy (V0, 1.84 ± 0.61 versus V1, 2.22 ± 0.55, *p* = 0.040) but dropped significantly after the wash-out period (V1, 2.22 ± 0.55 versus V2, 1.67 ± 0.57, *p* = 0.019; Table 1 and Fig. 2).

**Table 1:** Comparison of patient characteristics at different visits within study groups (*p value at V0 *vs* V1; **p value for V2 *vs V1)*

Variables	Control (*n* = 9)					Warm white light (*n* = 13)				
Sex (male/female)	3/6					6/7				
Age (years)	41.7 ± 6.3					39.5 ± 7.3				
Current or past/never smokers	1/8					2/11				
	V0	V1	V2	*p	**p	V0	V1	V2	*p	**p
Fasting glucose (mg/dl)	83.0 ± 4.1	89.5 ± 18.3	84.7 ± 3.2	0.335	0.057	86.1 ± 9.9	88.3 ± 15.3	85.0 ± 11.1	0.635	0.410
Fasting Insulin (µU/mL)	8.5 ± 4.1	8.3 ± 4.9	8.4 ± 5.0	0.682	0.699	8.1 ± 6.2	8.3 ± 5.8	8.2 ± 5.3	0.544	0.672
OGTT glucose (AUC)	251 ± 64	247 ± 61	260 ± 84	0.861	0.344	244 ± 51	255 ± 56	252 ± 36	0.778	0.331
OGTT Insulin (AUC)	239 ± 128	347 ± 249	378 ± 375	0.675	1.00	157 ± 105	320 ± 255	296 ± 165	0.002	0.695
HOMA-IR	2.1 ± 1.2	2.5 ± 2.0	2.7 ± 1.2	0.336	0.530	2.3 ± 1.4	2.3 ± 1.8	2.3 ± 1.6	0.638	0.875
HbA1c (%)	5.1 ± 0.0	5.0 ± 0.0	5.0 ± 0.0	0.470	0.577	5.2 ± 0.0	5.1 ± 0.0	5.2 ± 0.0	0.134	0.138
Triglycerides (mg/dl)	81.6 ± 52.9	84.2 ± 58.2	96.8 ± 88.1	0.331	0.588	88.5 ± 29.7	102.8 ± 43.5	72.2 ± 23.4	0.258	0.069
Total cholesterol (mg/dl)	194.2 ± 26.8	187.3 ± 29.0	192.2 ± 41.9	0.422	0.654	187.2 ± 33.5	185.7 ± 26.8	176.4 ± 33.5	0.875	0.084
LDL cholesterol (mg/dl)	127.3 ± 33.0	118.5 ± 25.0	117.3 ± 29.5	0.694	0.683	118.5 ± 31.0	106.7 ± 35.8	111.8 ± 29.9	0.593	0.258
HDL cholesterol (mg/dl)	59.2 ± 17.1	58.2 ± 16.0	56.1 ± 16.5	0.479	0.969	57.8 ± 12.6	56.6 ± 14.2	57.3 ± 16.7	0.220	0.850
CRP (mg/dl)	0.19 ± 0.3	0.12 ± 0.11	0.11 ± 0.04	0.196	0.562	0.20 ± 0.24	0.23 ± 0.21	0.16 ± 0.22	0.414	0.480
SBP (mmHg)	103.1 ± 17.3	114.3 ± 16.3	110.0 ± 16.9	0.145	0.904	107.5 ± 17.7	113.5 ± 13.6	112.5 ± 15.0	0.066	0.821
DBP (mmHg)	73.1 ± 13.6	75.0 ± 8.8	76.8 ± 5.9	1.003	0.183	74.3 ± 10.0	72.1 ± 9.3	74.6 ± 7.7	0.588	0.226
BMI	24.2 ± 3.9	24.0 ± 3.9	24.4 ± 3.8	0.311	0.065	25.9 ± 3.7	26.0 ± 3.8	25.9 ± 3.9	0.575	0.838
Waist circumference (cm)	88.6 ± 12.7	88.0 ± 12.3	88.1 ± 13.3	0.631	0.634	87.8 ± 10.8	87.8 ± 9.9	88.1 ± 8.9	0.825	0.531
HS CRP (mg/L)	0.45 ± 1.01	0.65 ± 1.32	0.13 ± 0.21	0.332	0.772	0.25 ± 0.24	0.21 ± 0.20	0.22 ± 0.21	0.672	0.771
PSQI global score (score 1–4/5–21)	3/5	2/6	2/6	0.421	0.999	3/10	3/10	2/11	0.999	0.562
REV-ERBα mRNA (AU)	2.01 ± 1.67	2.37 ± 1.54	1.64 ± 0.57	0.086	0.260	2.48 ± 1.54	2.07 ± 0.70	1.47 ± 0.96	0.048	0.074
B-MAL1 mRNA (AU)	1.01 ± 0.54	1.19 ± 0.50	1.00 ± 0.37	0.086	0.678	1.24 ± 1.86	0.95 ± 0.32	0.98 ± 0.79	0.109	0.177
REV-ERBα/BMAL1	1.99 ± 0.49	1.93 ± 0.55	1.64 ± 0.32	0.679	0.110	1.84 ± 0.61	2.22 ± 0.55	1.67 ± 0.57	0.040	0.019
IFN-γ (AU)	0.81 ± 0.87	0.78 ± 0.89	0.79 ± 0.87	0.833	0.879	0.88 ± 0.87	0.87 ± 0.86	0.89 ± 0.91	0.901	0.896
IL-1β (AU)	3.64 ± 5.89	3.97 ± 3.31	4.01 ± 4.76	0.619	0.845	6.45 ± 10.31	6.67 ± 5.77	5.99 ± 6.19	0.641	0.419

*BMI* body mass index, *SBP* systolic blood pressure, *DBP* diastolic blood pressure, *HOMA-IR* homeostatic model assessment of insulin resistance, *OGTT* oral glucose tolerance test, *AUC* area under the curve, *HbA1c* glycated hemoglobin, *HDL* high-density lipoprotein, *LDL* low-density lipoprotein, *CRP* C-reactive protein, *PSQI* Pittsburgh sleep quality index questionnaire, *IFN-γ* interferon-γ, *IL-1β* interleukin-1β, *AU* arbitrary unit

## Discussion

Exposure to direct sunlight enables organisms to synchronize their biological systems to the external world. Circadian signaling pathways controlled by a series of transcriptional/translational feedback cycles have been identified in virtually all eukaryotic cells. The circadian transcriptional activators, BMAL1 and CLOCK, regulate the expression of thousands of transcripts. These genes also control the expression of their own repressors, REV-ERBα and REV-ERBβ, thereby creating a form of negative feedback control on circadian gene expression. As a consequence of this mechanism, the internal clock of NSWs tends to be desynchronized as they are exposed to only a limited amount of light of the appropriate solar spectrum [3]. To address this deficit, we exposed a group of active rotating NSWs to a source of warm white light for a short period each day (i.e., 30 min each morning for 12 consecutive weeks). Bright light therapy is currently in wide use for the treatment of depression as well as sleep and other neurological disorders [17, 18]. However, in our study, we did find no significant changes in glucose metabolism, insulin resistance, lipid levels, or inflammation. The lack of any clinical effect of light therapy is somehow unexpected. In fact, in a recent publication derived from the EuRhythDia project, we reported that a group of NSWs undergoing 12 weeks of light therapy significantly showed an improvement of diurnal blood pressure control, a decrease in serum glucose during OGTT and a reduction in plasma metanephrine and nor-metanephrine levels [19]. However, the participants of two studies were markedly different. In fact, respect to the above-mentioned study, the number of participants undergoing light therapy in our study was lower (*n* = 24 versus *n* = 13), whereas the mean age and the proportion of female were higher (36 ± 13 vs 39.5 ± 7.3 and 29% versusvs 54%, respectively).

On contrast, our findings revealed significant modulation of both REV-ERBα expression and the REV-ERBα/BMAL1 mRNA ratio in PBMCs isolated from otherwise healthy rotating NSWs in our experimental group. Interestingly, the role of REV-ERBα in inflammation process has been reported both in human rodent experimental models [20]. In fact, REV-ERBα regulates the timing of NLRP3 expression and production of inflammatory cytokines by macrophages, reducing the severity of peritoneal inflammation and fulminant hepatitis.

Thus, although individual gene expression levels may differ between individuals, the REV-ERBs/BMAL1 ratio might be explored further as an indicator of desynchronization and, more importantly, resynchronization of the master circadian clock due to sleep disturbances and aberrant and prolonged exposure to artificial light [21].

Notably, several recent findings highlight the roles of BMAL1 and REV-ERBα as regulators of disease severity in patients diagnosed with acute respiratory syndrome-Coronavirus-2 (SARS-CoV-2) infection [22]. In these cases, altered expression of circadian clock genes results in increased disease severity largely via increases in the extent of pathological pulmonary inflammation [23]. This is of particular importance to the health care workers, including those featured in our study. Although health care workers represent only 3% of the population, they represented 14% of all cases of Coronavirus disease-2019 (COVID-19) [24].

The role of bright light therapy in restoring appropriate circadian rhythms has been reported in studies of patients diagnosed with both Alzheimer’s and Parkinson’s disease [25]. While the mechanisms remain unclear, bright light therapy may provide relief by targeting absolute and relative expression levels of circadian clock genes**.** Results from previous studies performed in both humans and rodent experimental models, suggested that nocturnal light exposure has a profound impact on pathways linked to glucose metabolism, including melatonin biosynthesis [26, 27] and corticosteroid rhythmicity [28, 29]. However, the short treatment period featured in our study might conceivably result in altered REV-ERBα/BMAL1 mRNA ratios despite no discernible impact on glucose metabolism or quality of sleep.

Our study has several clear limitations. This work is a pilot study with only a few participants and a limited time frame. Thus, it may be underpowered for clinical endpoints, including modulation of glucose metabolism. Prolonged exposure to bright light may ultimately have a more substantial influence on gene expression, sleep, inflammation, and metabolism. Nonetheless, to the best of our knowledge, this is the first clinical study to highlight an important biological parameter as the direct or indirect target of an effective non-pharmacological strategy designed to address these conditions.

In conclusion, the results of our work reveal a measurable and significant effect of warm white light on the REV-ERBα/BMAL1 mRNA ratio in PMBCs of rotating NSWs. Collectively, our findings suggest that this straightforward and non-pharmacological strategy might be used to restore disruptions to the circadian clock. Future studies might address the effects of bright light in a larger population of NSWs using longer exposure periods or crossover study design to assess critical clinical variables including glucose dysregulation, sleep, and overall quality of life.
